# Comparison of Stromal Tumor-Infiltrating Lymphocyte (sTIL) Levels and Clinicopathological Features in Neoadjuvant-Naive HER2-Low and HER2-Negative Primary Breast Cancers

**DOI:** 10.3390/medicina62050826

**Published:** 2026-04-27

**Authors:** Mümin Emiroğlu, Esra Canan Kelten Talu, Cem Karaali, Olçun Ümit Ünal, Mihriban Erdoğan

**Affiliations:** 1 Department of Pathology, Elazığ Fethi Sekin City Hospital, Elazığ 23100, Turkey; 2 Department of Pathology, UHS Izmir Faculty of Medicine, Izmir Tepecik Education and Research Hospital, Izmir 35020, Turkey; ecanankelten@gmail.com; 3 Department of General Surgery, UHS Izmir Faculty of Medicine, Izmir Tepecik Education and Research Hospital, Izmir 35020, Turkey; cemkaraali@gmail.com; 4 Department of Medical Oncology, UHS Faculty of Medicine, Izmir City Hospital, Izmir 35540, Turkey; drolcun@hotmail.com; 5 Department of Radiation Oncology, UHS Faculty of Medicine, Izmir City Hospital, Izmir 35540, Turkey; mihribankocak@hotmail.com

**Keywords:** breast cancer, HER2-low, stromal tumor-infiltrating lymphocytes, prognosis, tumor microenvironment, antibody-drug conjugates

## Abstract

*Background and Objective*: The clinical success of novel antibody-drug conjugates has led to the identification of a new subgroup within traditionally HER2-negative breast cancers, termed ‘HER2-low.’ The aim of this study was to investigate the clinicopathological differences between HER2-low and HER2-negative groups in neoadjuvant-naive primary breast cancer patients, with a specific focus on stromal tumor-infiltrating lymphocyte (sTIL) density. *Materials and Methods*: The study included 731 neoadjuvant-naive invasive breast cancer patients. Tumors were classified as HER2-negative (IHC 0) and HER2-low (IHC 1+ or 2+/ISH-negative). sTIL levels were evaluated following the International TILs Working Group guidelines. *Results*: The HER2-low group (38.7%) demonstrated significantly higher histological grade (*p* = 0.033) and higher sTIL density (*p* = 0.006) compared to the HER2-negative group. A stepwise increase in sTIL rates was observed parallel to the HER2 immunohistochemical score (0 → 1+ → 2+) (*p* = 0.015). The HER2-low/hormone receptor (HR)-negative subgroup exhibited the highest sTIL density (median 35%). No statistically significant difference in overall or disease-free survival was found between the groups. *Conclusions*: HER2-low breast cancers were associated with a more immunogenic tumor microenvironment compared to HER2-negative tumors. This robust immune infiltration may offset the higher histological grade observed in the HER2-low cohort, potentially explaining the comparable survival outcomes. These findings provide a biological rationale for exploring the synergy between novel antibody–drug conjugates and immune checkpoint inhibitors, particularly in the highly immunogenic HER2-low/HR-negative subgroup.

## 1. Introduction

Breast cancer is a highly heterogeneous group of diseases in terms of biological behavior and treatment responses, and it is traditionally classified based on hormone receptor (HR) and Human Epidermal Growth Factor Receptor 2 (HER2) status [1]. In clinical practice, HER2 status is determined according to guidelines from the American Society of Clinical Oncology and the College of American Pathologists (ASCO/CAP); tumors with an immunohistochemistry (IHC) score of 3+ or showing gene amplification by in situ hybridization (ISH) with an IHC score of 2+ are considered “HER2-positive,” while others are considered “HER2-negative [2,3]”. HER2-positive breast cancers account for approximately 15–20% of all cases, while the majority of patients fall into the HER2-negative group, which does not benefit from traditional anti-HER2 therapies [1,2].

However, the development of new generation antibody–drug conjugates (ADCs) in recent years, especially trastuzumab deruxtecan (T-DXd), has challenged the current classification system by demonstrating clinical activity in tumors that do not overexpress HER2 but do express low levels of HER2 protein [1,2,4]. In light of these developments, the term ‘HER2-low’ has been introduced for tumors previously classified as “HER2-negative’ but with IHC 1+ or IHC 2+/ISH-negative; tumors that show no staining at all are classified as ‘HER2-zero/null’ [2,3,5]. The question of whether the HER2-low group, which accounts for approximately 45–55% of all breast cancers, is a distinct entity from the HER2-negative group both clinically and biologically remains one of the most debated topics in current oncology [1,3,6].

The data in the literature suggest that HER2-low tumors exhibit a higher rate of hormone receptor positivity (HR+), lower tumor grade, and a lower proliferation index (Ki-67) compared to HER2-negative tumors [1,2,4,7]. Molecular-level Prediction Analysis of Microarray 50 (PAM50) analyses indicate that the majority of HER2-low tumors are of the Luminal subtype and that HR status, rather than HER2 expression level, predominantly influences tumor biology [4,8]. However, there are conflicting results regarding the prognostic value of HER2-low status and its impact on response to chemotherapy, and the immunological characteristics of this group have not yet been fully elucidated [1,9,10].

Stromal tumor-infiltrating lymphocytes (sTILs), a fundamental component of the tumor microenvironment, are recognized by the latest World Health Organization (WHO) guidelines as a biomarker with proven predictive and prognostic value, especially in triple-negative and HER2-positive breast cancers [9,11]. However, studies on the immune microenvironment and sTIL density in HER2-low breast cancers are limited. Some studies have reported that HER2-low tumors may have lower sTIL density than HER2-negative tumors, whereas others have found no significant difference [9,12]. For example, Fernandes et al. reported that the sTIL levels in HER2-low tumors were significantly lower than in HER2-positive tumors but similar to those in the HER2-negative group [12].

Most current studies have focused on residual disease or metastatic processes after neoadjuvant chemotherapy (NACT), and data comparing sTIL levels with clinicopathological features in the tumor microenvironment at diagnosis, without the modifying effect of systemic therapy, are limited [12,13,14]. Evaluations in patients who have not received NACT are critical for understanding the biology of HER2-low tumors without the confounding effects of treatment.

Despite the emerging clinical significance of HER2-low breast cancer, the specific nature of its immune microenvironment, particularly in neoadjuvant-naive settings, remains insufficiently characterized. Most existing studies include patients treated with neoadjuvant therapies, which may alter baseline sTIL levels. Therefore, this study aims to investigate the association between HER2-low status and sTIL density in a pure, treatment-naive cohort. Our primary hypothesis is that HER2-low tumors exhibit a distinct immunological profile that is independent of other established clinicopathological variables.

## 2. Materials and Methods

This study was designed as a retrospective, hospital-based cross-sectional study. To minimize selection bias, a consecutive sampling method was employed. A total of 1193 primary invasive breast cancer cases diagnosed and treated at Tepecik Training and Research Hospital between 2011 and 2018 were initially screened through the hospital information system. The inclusion criteria were: (1) histologically confirmed invasive breast carcinoma; (2) availability of full-face hematoxylin and eosin (H&E) stained slides and formalin-fixed paraffin-embedded (FFPE) blocks for re-evaluation; and (3) presence of complete clinical follow-up data. The exclusion criteria were: (1) patients who received neoadjuvant chemotherapy (NACT), to avoid treatment-induced alterations in the tumor microenvironment and sTIL density; (2) cases with insufficient pathological material for HER2 and sTIL assessment; and (3) patients with incomplete medical records. After applying these criteria, 731 patients were ultimately included in the final study cohort. The average follow-up duration for these patients was 88.7 months.

The evaluated parameters included patient age, tumor size, histological type, nuclear grade, histological grade, stromal TIL percentage, presence of tumor necrosis, ER staining intensity and percentage, PgR staining intensity and percentage, HER2 immunohistochemistry score, Ki-67 proliferation index, presence of metastatic lymph nodes, distant metastasis, clinical stage, type of surgery, overall survival, and disease-free survival parameters.

HER2 and HR Status Evaluation: HER2 status was assessed using immunohistochemistry (IHC). The FDA-approved A0485 polyclonal antibody was used for HER2 immunohistochemical staining. The HER2-low group was recorded as cases with an IHC score of 1+ or 2+/ISH negative. In the immunohistochemical assessment of HER2, the guidelines of the American Society of Clinical Oncology and the American Pathologists (ASCO/CAP) were considered [15]. For hormone receptor (ER/PgR) status, nuclear expression levels of 1% or more were considered positive according to the ASCO/CAP guidelines [16].

**Figure 1:**
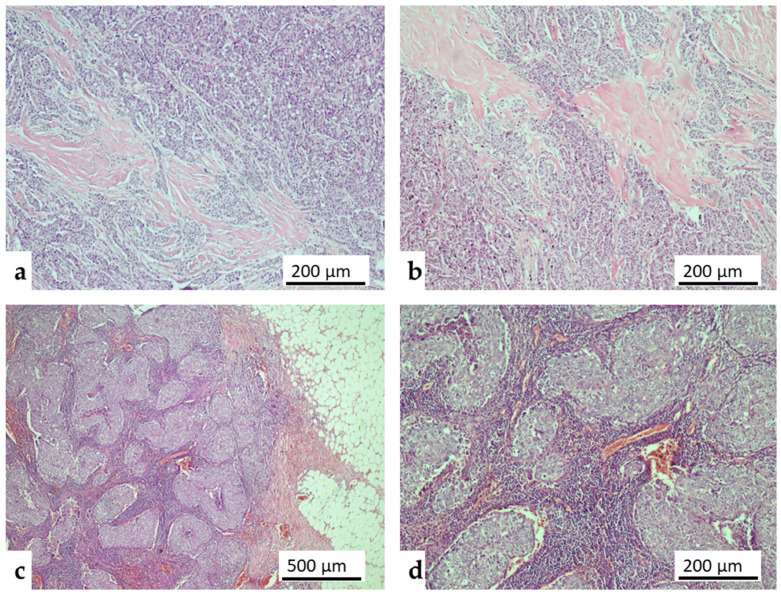
Representative microscopic images of stromal tumor-infiltrating lymphocytes (sTILs) in primary breast cancer (Hematoxylin and Eosin stain). (**a**,**b**) Tumors exhibiting a low sTIL phenotype, characterized by sparse mononuclear inflammatory cells within the invasive tumor stroma (Original magnification: 100×). (**c**,**d**) Tumors displaying a high sTIL phenotype, demonstrating dense and prominent lymphocytic infiltration closely surrounding the invasive tumor nests (Original magnification: 40× and 100×).

Stromal TIL percentage: In the assessment of the Stromal TIL score, the recommendations of the International TIL Consensus Working Group were taken into account [17] (Figure 1). Accordingly, TIL was evaluated only in the invasive tumor stroma. When providing the ratio, the area of the stromal tissue was used as the denominator. All mononuclear cells (lymphocytes and plasma cells) were evaluated, while polymorphonuclear leukocytes were excluded from scoring [17].

Data Analysis: Data were analyzed using IBM SPSS Statistics 26 (IBM Corp., Armonk, NY, USA). Descriptive statistics were presented as mean ± standard deviation for normally distributed continuous variables, median (interquartile range) for non-normally distributed variables, and frequency (percentage) for categorical variables. The Mann–Whitney U test or independent samples *t*-test was used to compare continuous variables between HER2-negative and HER2-low groups, while the Chi-square test was used for categorical comparisons. A stepwise ‘immunological gradient’ across HER2 scores (0, 1+, 2+) was assessed using the Kruskal–Wallis test. Survival analyses were performed using the Kaplan–Meier method and the Log-rank test. To identify independent predictors of high sTIL density (>10%), a multivariable logistic regression analysis was conducted. Furthermore, multivariable Cox proportional hazards regression models were utilized to evaluate the independent prognostic significance of HER2-low status on disease-free survival (DFS) and overall survival (OS), adjusting for potential confounders such as age, tumor size, histological grade, and hormone receptor status. A *p*-value of less than 0.05 was considered statistically significant.

GenAI (Gemini (Google, version 3.1 Pro)) was used for linguistic refinement, structural organization, and translating draft sections of the manuscript to ensure academic English standards. The final intellectual content and pathological evaluations were performed solely by the authors.

## 3. Results

The baseline clinicopathological characteristics of the 731 patients included in the study are summarized in Table 1.

Compared to the HER2-negative group, HER2-low tumors exhibited significantly higher histological grades (*p* = 0.033) and greater sTIL density (*p* = 0.006) (Table 2, Figure 1).

Subgroup analysis by HER2 and HR expression revealed that the HER2-low/HR-negative phenotype had the highest median sTIL density (35%), along with the largest tumor size and the highest nuclear and histological grades (Table 3).

A high sTIL density (>10%) was significantly associated with HER2-low status, which accounted for **48.0%** of the high sTIL cases, compared to **34.5%** in the HER2-negative group (***p*** **< 0.001**) (Table 4).

**Figure 2:**
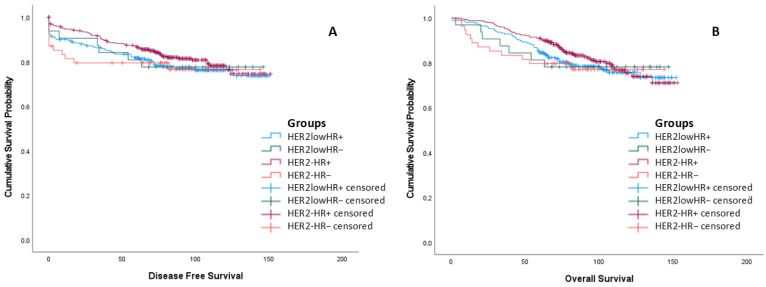
Survival outcomes categorized by HER2 and hormone receptor (HR) status. (**A**) Kaplan–Meier curves for disease-free survival (DFS) and (**B**) overall survival (OS). Despite the biological heterogeneity observed between groups, no statistically significant differences were found in mean DFS (*p* = 0.609) or mean OS (*p* = 0.697) across the four defined subgroups. Censored cases are indicated by “+” symbols on the survival curves. Statistical significance was determined using the Log-rank test.

The disease-free and overall survival times of the groups do not differ statistically (Table 5, Figure 2).

To determine whether HER2-low status is an independent prognostic factor, a multivariable Cox proportional hazards regression model was performed adjusting for age, tumor size, histological grade, hormone receptor status, and sTIL density. The analysis revealed that only patient age (HR = 1.034, *p* < 0.001) and tumor size (HR = 1.138, *p* < 0.001) were significant independent predictors of survival. Importantly, HER2-low status was not significantly associated with worse survival outcomes when adjusted for these covariates (HR = 1.247, 95% CI: 0.894–1.740, *p* = 0.194), confirming the univariate Kaplan–Meier findings (Table 6).

Similarly, the multivariable Cox regression model for overall survival (OS) yielded consistent results. Only patient age (HR = 1.036, *p* < 0.001) and tumor size (HR = 1.162, *p* < 0.001) emerged as independent prognostic factors for overall survival. HER2-low status did not show a statistically significant association with worse overall survival when adjusted for the relevant clinicopathological covariates (HR = 1.238, 95% CI: 0.886–1.729, *p* = 0.211) (Table 7).

**Figure 3:**
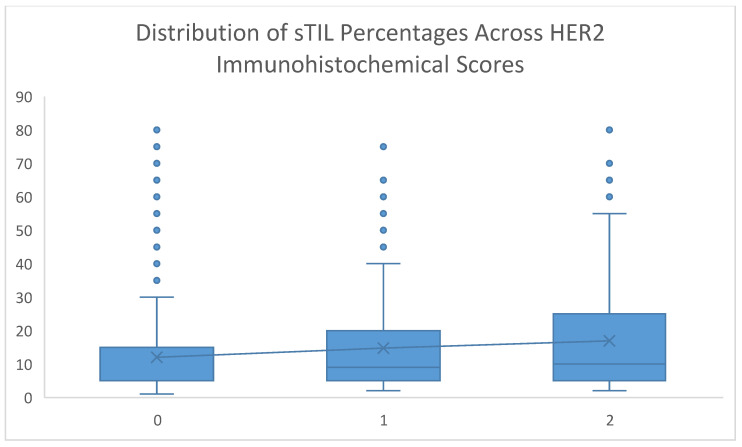
Distribution of sTIL percentages across HER2 immunohistochemical scores. The box-plot illustrates a stepwise ‘immunological gradient’, with a gradual increase in the median density of stromal tumor-infiltrating lymphocytes (sTILs) as the HER2 score progresses from 0 to 1+ and 2+ (*p* = 0.015). The horizontal black lines within the boxes represent the median values.

Representative histopathological images of tumors with low and high sTIL density are presented in Figure 1. A stepwise ‘immunological gradient’ was observed within the cohort, with median sTIL ratios gradually increasing in tandem with higher HER2 immunohistochemical scores (0 → 1+ → 2+) (*p* = 0.015) (Table 8, Figure 3).

To account for potential confounding factors, a multivariable logistic regression analysis was performed to identify independent predictors of high sTIL density (>10%). The model included HER2 status, histological grade, hormone receptor (HR) status, and Ki-67 index. The results demonstrated that HER2-low status remained a significant independent predictor of high sTIL density, with an odds ratio of 1.568 (95% CI: 1.076–2.286, *p* = 0.019), even after adjusting for histological grade, HR status, and proliferation index (Table 9). As expected, higher histological grade (*p* < 0.001) and Ki-67 index (*p* < 0.001) were also independent predictors of high sTILs, while HR positivity was associated with lower sTIL levels (OR = 0.252, *p* < 0.001).

The high sTIL phenotype (>10%) was significantly associated with younger age, higher nuclear and histological grades, elevated Ki-67 proliferation index, and the presence of tumor necrosis (*p* < 0.05 for all parameters) (Table 10).

## 4. Discussion

In this study, the clinicopathological features and immune microenvironments of HER2-low and HER2-negative tumors were comparatively analyzed in a large cohort of primary breast cancer patients who did not receive neoadjuvant therapy. The data revealed that the HER2-low group exhibited a more aggressive phenotype, characterized by a higher histological grade and a significantly higher sTIL density than the HER2-negative group. The most striking result of our study was the statistically significant ‘gradient’ increase in sTIL levels, parallel to the increase in HER2 expression (from 0 to 2+). Notably, the HER2-low/HR-negative subgroup with the highest immune infiltration suggests that this group biologically differs from the HER2-negative group and has potential as an immunological target; however, these biological differences did not translate into significant changes in survival outcomes (OS and DFS) within the current follow-up period.

The study cohort has a wide age range and stage distribution, which can represent the general breast cancer population. The presence of a metastatic patient group enables evaluation of immune microenvironment analyses in the context of advanced-stage disease.

In the comparison between the HER2-low and HER2-negative groups (Table 2), the HER2-low group has a higher histological grade (*p* = 0.033) and higher sTIL levels (*p* = 0.006). These findings contradict the general consensus in the literature. Many studies have reported that HER2-low tumors have lower Ki-67 and lower grade compared to HER2-negative tumors [18,19,20]. In our study, however, the HER2-low group exhibits a more aggressive phenotype (higher grade and higher sTIL), highlighting the heterogeneity within the HER2-low category. Furthermore, contrary to the findings of Fernandes et al. [12], we observed higher sTIL levels in the HER2-low group. This result supports the hypothesis that even low levels of HER2 protein can induce immunogenicity (immune response) [21], suggesting that HER2 protein on the cell surface serves as an antigenic stimulus that triggers T-cell infiltration.

While data in the literature define HER2-low/HR-negative tumors as having lower proliferation and more ‘indolent’ clinical features than the HER2-negative group [2,22,23], our study identified this subgroup as having a more aggressive and immunogenic phenotype, characterized by higher tumor grade and dense lymphocytic infiltration. This finding aligns with recent, limited studies highlighting the poor prognostic features of this group [24,25].

In the literature, the prognostic value of sTILs has generally been evaluated using cut-off values such as 10%, 20%, or 50% [26]. The use of a 10% threshold in our study and the clustering of patients with HER2-low status above this threshold are consistent with the strong association between high sTIL and HER2 observed in the study by Kınıkoğlu and colleagues [27]. This situation may serve as a clue that when lymphocyte-rich stroma is observed in pathological assessment, the pathologist should pay closer attention to HER2 staining.

Despite differences observed in biological markers (Grade, sTIL, Ki-67) in subgroup analyses, the lack of statistically significant differences in survival times (OS and DFS) (*p* > 0.05) is noteworthy (Table 5). This situation is consistent with the literature data [1,5,6,8,22,28]. Large-scale studies by Zhang and colleagues, Rodrigues and colleagues, and meta-analyses have not found a significant survival difference between HER2-low and HER2-negative patients under standard treatment [5,19,29]. The similar survival outcomes between the HER2-low and HER2-negative groups may be due to the protective immune response conferred by high sTIL levels in the former or to the effectiveness of standard treatments.

As HER2 expression increases (0 → 1 → 2), the sTIL ratio also gradually increases, which is an important finding (Table 6). Studies in the literature indicate that an increase in HER2 expression is associated with changes in biological behavior, even in the absence of gene amplification [21,30]. In our study, a positive, gradual relationship was found between HER2 protein expression level and stromal lymphocyte infiltration. In particular, tumors with HER2 Score 2+ exhibit significantly higher immune response (sTIL) than those with Score 0, indicating an immunological gradient within the HER2-low spectrum.

A high sTIL level (>10%), young age, high histological grade, increased proliferation (Ki-67), and tumor necrosis characterize an aggressive tumor phenotype (Table 7). It appears that the immune system develops a stronger response against biologically most active and rapidly proliferating tumors [31]. The observed increase in sTILs within HER2-low tumors may be attributed to a higher neoantigen load or distinct antigenic stimuli associated with low-level HER2 expression, which could potentially trigger a more robust anti-tumor immune response.

In alignment with the recent updates in the WHO Classification of Breast Tumours, this study emphasizes the critical clinical necessity of routinely evaluating and reporting the percentage of stromal tumor-infiltrating lymphocytes (sTILs) in daily pathological practice [10]. As the WHO highlights, morphological sTIL assessment should increasingly be incorporated into pathology reports as a routine prognostic factor, akin to established parameters such as tumor size, histological grade, or nodal status [10]. The observation of a more robust immune microenvironment in HER2-low breast cancers, particularly within the HR-negative subgroup, warrants deeper biological consideration. While our observational study design precludes establishing direct causality, it is plausible that even low-level HER2 protein expression may act as a continuous antigenic stimulus, promoting the recruitment of sTILs and dynamically reshaping the tumor microenvironment (TME). In this context, the PD-1/PD-L1 axis is of paramount clinical relevance. It is well-documented that tumors with elevated sTILs frequently upregulate immune checkpoint molecules as a mechanism of adaptive immune resistance. Therefore, the highly immunogenic HER2-low/HR-negative subgroup identified in our cohort represents a compelling candidate for synergistic therapeutic strategies. Specifically, combining next-generation antibody–drug conjugates (ADCs) with immune checkpoint inhibitors (e.g., anti-PD-1/PD-L1 agents) could potentially exploit this active immune microenvironment, offering a robust biological rationale for future targeted clinical trials.

This study has certain limitations that should be acknowledged. First, the retrospective, single-center design may limit the generalizability of our findings to more diverse populations and introduces potential retrospective bias. Second, HER2-low status was determined solely via immunohistochemistry (IHC) without advanced gene expression profiling (e.g., PAM50), which could provide deeper molecular insights into subtype distribution. Furthermore, the inherent subjectivity of IHC, particularly in distinguishing between IHC 0 and IHC 1+, remains a well-known challenge in HER2-low classification, although this was mitigated in our study by having all slides evaluated by a single expert pathologist. Finally, while our cohort was neoadjuvant-naive, potential heterogeneity in adjuvant treatment regimens could have influenced long-term survival outcomes.

## 5. Conclusions

In conclusion, our findings demonstrate that HER2-low primary breast cancers possess a distinct, more active immune microenvironment compared to HER2-negative tumors, independent of neoadjuvant therapy effects. The stepwise increase in sTILs correlated with elevated HER2 expression suggests a potential immunogenic role for low-level HER2 protein. While these biological differences did not translate into survival advantages within our observational cohort, they highlight the HER2-low/HR-negative phenotype as a biologically active entity. Recognizing this robust immune profile provides a strong rationale for future clinical trials aimed at evaluating targeted synergistic strategies, such as ADCs combined with immunotherapy, tailored specifically for this patient population. Furthermore, our study reinforces the clinical necessity of routinely evaluating and reporting sTIL levels in daily pathological practice.

## Figures and Tables

**Table 1:** Descriptive statistics of patients (N = 731).

Variables	Statistics
**Age** (*years*)	
$\bar{x}$ ± *ss*	56.4 ± 12.8
*min–max*	28–93
**Stage**, *n* (%)	
N/A	44 (6.0)
1A	180 (24.6)
2A	226 (30.9)
2B	119 (16.3)
3B	1 (0.1)
3C	32 (4.4)
4	129 (17.6)
**Histological Type**, *n* (%)	
NST	653 (89.3)
Lobular	58 (7.9)
Papillary	16 (2.2)
Mucinous	4 (0.5)
**Survival**, *n* (%)	
Deceased	148 (20.2)
Alive	583 (79.8)
**Surgical Type**, *n* (%)	
BCS + SLNB	521 (71.3)
BCS	27 (3.7)
MRM	125 (17.1)
SM	58 (7.9)

*n*: Sample size, %: Percentage value, $\bar{x}$: Mean, *ss*: Standard deviation. BCS: Breast-Conserving Surgery, SLNB: Sentinel Lymph Node Biopsy, MRM: Modified Radical Mastectomy, SM: Simple Mastectomy. N/A: Not Available. Exact clinical/pathological staging could not be retrieved for 44 patients due to missing historical records.

**Table 2:** Comparison of HER2-low and HER2-negative groups.

	HER2		Test Statistics	
	HER2-Negative *n* = 448	HER2-Low *n* = 283	Test Value	*p*-Value
**Age** (*years*)	57.0 ± 12.9	55.4 ± 12.7	1.667	0.096 ^†^
**Tumor size** (cm)	2.77 ± 1.83	2.89 ± 1.54	0.928	0.357 ^†^
**Nuclear Grade**, *n* (%)				
1	33 (7.4)	12 (4.2)		
2	290 (64.7)	180 (63.6)	3.849	0.146 ^‡^
3	125 (27.9)	91 (32.3)		
**Histological Grade**, *n* (%)				
1	27 (6.0)	11 (3.9)		
2	301 (67.2)	172 (60.8)	6.842	**0.033** ^‡^
3	120 (26.8)	100 (35.3)		
**Necrosis**, *n* (%)				
None	373 (83.3)	230 (81.3)	0.474	0.491 ^‡^
Present	75 (16.7)	53 (18.7)		
**DCIS**, *n* (%)				
None	143 (31.9)	82 (29.0)	0.706	0.401 ^‡^
Present	305 (68.1)	201 (71.0)		
**Ki-67** (%)	15.0 (20.0)	20.0 (20.0)	1.950	0.051 ^&^
**Distant metastasis**, *n* (%)				
None	376 (83.9)	222 (78.7)	3.166	0.075 ^‡^
Present	72 (16.1)	60 (21.3)		
**sTIL** (%)	5 (10)	10 (15)	2.775	**0.006** ^&^

*n*: Number of cases, %: Column percentage, Numerical data are summarized as mean ± standard deviation or median (distance between quartiles, ^†^: *t*-test in independent samples, ^‡^: Pearson chi-square test, ^&^: Mann–Whitney U test. DCIS: Ductal Carcinoma In Situ; sTIL: Stromal Tumor-Infiltrating Lymphocytes.

**Table 3:** Comparisons according to HER2 and hormone receptor expression status.

	Groups				Test Statistics	
	HER2-Low/HR+ *n* = 251	HER2-Low/HR− *n* = 32	HER2-Negative/HR+ *n* = 394	HER2-Negative/HR− *n* = 54	Test Value	*p*-Value
**Age** (years)	55.5 ± 12.6	54.4 ± 12.9	57.1 ± 12.7	56.2 ± 14.2	1.085	0.355 *
**Tumor size** (cm)	2.78 ± 1.46 ^a^	3.75 ± 1.92 ^b^	2.75 ± 1.88 ^a^	2.92 ± 1.36 ^a^	3.450	**0.016** *
**Nuclear Grade**, *n* (%)					132.528	**<0.001**
1	12 (4.8) ^a^	0 (0.0) ^a^	33 (8.4) ^a^	0 (0.0) ^a^		
2	176 (70.1) ^a^	4 (12.5) ^b^	281 (71.3) ^a^	9 (16.7) ^b^		
3	63 (25.1) ^a^	28 (87.5) ^b^	80 (20.3) ^a^	45 (83.3) ^b^		
**Histological Grade**, *n* (%)					107.787	**<0.001 ** ^‡^
1	11 (4.4) ^a^	0 (0.0) ^a^	27 (6.9) ^a^	0 (0.0) ^a^		
2	168 (66.9) ^a^	4 (12.5) ^b^	287 (72.8) ^a^	14 (25.9) ^b^		
3	72 (28.7) ^a^	28 (87.5) ^b^	80 (20.3) ^a^	40 (74.1) ^b^		
**Necrosis**, *n* (%)						
None	220 (87.6) ^a^	10 (31.3) ^b^	347 (88.1) ^a^	26 (48.1) ^b^	88.345	**<0.001** ^‡^
Present	31 (12.4) ^a^	22 (68.7) ^b^	47 (11.9) ^a^	28 (51.9) ^b^		
**DCIS**, *n* (%)						
None	65 (25.9) ^a^	17 (53.1) ^b^	115 (29.2) ^a^	28 (51.9) ^b^	20.775	**<0.001** ^‡^
Present	186 (74.1) ^a^	15 (46.9) ^b^	279 (70.8) ^a^	26 (48.1) ^b^		
**Ki-67** (%)	15.0 (15.0) ^a^	40.0 (40.0) ^b^	15.0 (15.0) ^a^	40.0 (34.0) ^b^	**94.545**	**<0.001** **
**Distant metastasis**, *n* (%)						
None	205 (77.7)	27 (81.8)	343 (84.7)	44 (77.2)	6.045	0.109 ^‡^
Present	59 (22.3)	6 (18.2)	62 (15.3)	13 (22.8)		
**sTIL** (%)	5.0 (10.0) ^a^	35.0 (20.0) ^b^	5.0 (5.0) ^a^	22.5 (30.0) ^b^	**85.118**	**<0.001** **

*n*: Number of cases, %: Column percentage. Numerical data are summarized as mean ± standard deviation or median (interquartile range). MLN: Metastatic Lymph Node, TLN: Total Lymph Node, *: One-way ANOVA, ^‡^: Pearson chi-square test, **: Kruskal–Wallis analysis, the superscript letters ^a^ and ^b^ indicate differences between groups in each row. Groups sharing the same superscript are not statistically different.

**Table 4:** Comparison of HER2 groups with sTIL groups.

	HER2		Test Statistics	
	HER2-Negative *n* = 448	HER2-Low *n* = 283	Test Value	*p*-Value
**sTIL**				
≤10	329 (65.5)	173 (34.5)	**12.210**	**<0.001** ^‡^
>10	119 (52.0)	107 (48.0)		

*n*: Number of cases, %: Column percentage, ^‡^: Pearson chi-square test.

**Table 5:** Comparison of survival times according to HER2 and hormone receptor status.

					Test Statistics	
	HER2-Low/HR+ *n* = 251	HER2-Low/HR− *n* = 32	HER2-Negative/HR+ *n* = 394	HER2-Negative/HR− *n* = 54	Chi-Square Value	*p*-Value ^¥^
**Disease-free survival**	120.9 ± 3.5	119.4 ± 9.1	127.3 ± 2.5	113.4 ± 7.8	1.828	0.609
**Overall survival**	127.4 ± 2.9	122.1 ± 8.5	131.3 ± 2.2	117.5 ± 15.8	1.437	0.697

Data are summarized as mean ± standard error. ^¥^: Log-rank test.

**Table 6:** Multivariable Cox regression analysis for disease-free survival (DFS).

Variable	Hazard Ratio (HR)	95% Confidence Interval (CI)	*p*-Value
**Age (continuous)**	1.034	1.020–1.047	<0.001
**Tumor Size (continuous)**	1.138	1.075–1.206	<0.001
**Histological Grade**			0.573
Grade 3 (Reference)	1.00	-	-
Grade 1	0.681	0.284–1.636	0.391
Grade 2	0.848	0.582–1.236	0.391
**Hormone Receptor Status**			
Negative (Reference)	1.00	-	-
Positive	1.084	0.636–1.849	0.766
**sTIL Density**			
≤10% (Reference)	1.00	-	-
>10%	1.038	0.706–1.525	0.851
**HER2 Status**			
HER2-negative (Reference)	1.00	-	-
HER2-low	1.247	0.894–1.740	0.194

**Table 7:** Multivariable Cox regression analysis for overall survival (OS).

Variable	Hazard Ratio (HR)	95% Confidence Interval (CI)	*p*-Value
**Age (continuous)**	1.036	1.023–1.050	<0.001
**Tumor Size (continuous)**	1.162	1.096–1.232	<0.001
**Histological Grade**			0.771
Grade 3 (Reference)	1.00	-	-
Grade 1	0.752	0.313–1.808	0.524
Grade 2	0.903	0.619–1.318	0.598
**Hormone Receptor Status**			
Negative (Reference)	1.00	-	-
Positive	1.150	0.674–1.960	0.608
**sTIL Density**			
≤10% (Reference)	1.00	-	-
>10%	1.078	0.735–1.582	0.701
**HER2 Status**			
HER2-negative (Reference)	1.00	-	-
HER2-low	1.238	0.886–1.729	0.211

**Table 8:** Comparison of sTIL percentages according to HER2 scores.

	HER2 score			Test Statistics	
	0 *n* = 448	1 *n* = 148	2 *n* = 135	Test Value	*p*-Value
**sTIL**	5 (10) ^a^	9 (15) ^ab^	10 (20) ^b^	8.353	0.015 **

The numerical data is summarized as the median (interquartile range (IQR)). **: Kruskal–Wallis analysis, the superscripts ^a^ and ^b^ in each row indicate between-group differences.

**Table 9:** Multivariable logistic regression analysis of factors associated with high sTIL density (>10%).

Variable	Odds Ratio (OR)	95% Confidence Interval (CI)	*p*-Value
**HER2 Status (Low vs. Zero)**	1.568	1.076–2.286	0.019
**Histological Grade**	-	-	<0.001
**HR Status (Positive vs. Negative)**	0.252	0.137–0.461	<0.001
**Ki-67 Index**	1.021	1.009–1.033	<0.001

**Table 10:** Comparisons by sTIL percentage.

	sTIL		Test Statistics	
	≤10 *n* = 502	>10 *n* = 229	Test Value	*p*-Value
**Age** (*years*)	57.1 ± 12.6	54.8 ± 13.2	2.327	**0.020** ^†^
**Tumor size** (cm)	2.76 ± 1.84	2.94 ± 1.42	1.367	0.172 ^†^
**Nuclear Grade**, *n* (%)				
1	41 (8.2) ^a^	4 (1.7) ^b^		
2	369 (73.5) ^a^	101 (44.1) ^b^	99.968	**<0.001** ^‡^
3	92 (18.3) ^a^	124 (54.1) ^b^		
**Histological Grade**, *n* (%)				
1	34 (6.8) ^a^	4 (1.7) ^b^		
2	368 (73.3) ^a^	105 (45.9) ^b^	81.092	**<0.001** ^‡^
3	100 (19.9) ^a^	120 (52.4) ^b^		
**Necrosis**, *n* (%)				
None	457 (91.0)	146 (63.8)	81.027	**<0.001** ^‡^
Present	45 (9.0)	83 (36.2)		
**DCIS**, *n* (%)				
None	137 (27.3)	88 (38.4)	9.155	**0.002** ^‡^
Present	365 (72.7)	141 (61.6)		
**Ki-67** (%)	15 (10)	25 (25)	7.861	**<0.001** ^&^
**Distant metastasis**, *n* (%)				
None	416 (83.0)	182 (79.5)	1.343	0.246 ^‡^
Present	85 (17.0)	47 (20.5)		

*n*: Number of cases, %: Column percentage, Numerical data are summarized as mean ± standard deviation or median (distance between quartiles, ^†^: *t*-test in independent samples, ^‡^: Pearson chi-square test, ^&^: Mann–Whitney U test. The superscript letters indicate the statistical tests used for the comparisons: ^a^: Independent samples *t*-test; ^b^: Pearson chi-square test

## Data Availability

The data presented in this study are available on request from the corresponding author. The data are not publicly available due to privacy restrictions.

## Abbreviations

The following abbreviations are used in this manuscript:

sTIL	Stromal Tumor-Infiltrating Lymphocytes
HER2	Human Epidermal Growth Factor Receptor 2
ADC	Antibody-Drug Conjugate
NACT	Neoadjuvant Chemotherapy
IHC	Immunohistochemistry
ISH	In Situ Hybridization
ASCO	American Society of Clinical Oncology
CAP	College of American Pathologists
HR	Hormone Receptor
DFS	Disease-Free Survival
OS	Overall Survival
OR	Odds Ratio
ER	Estrogen Receptor
PgR	Progesterone Receptor
DCIS	Ductal Carcinoma In Situ
PAM50	Prediction Analysis of Microarray 50
T-DXd	Trastuzumab Deruxtecan
BCS	Breast-Conserving Surgery
SLNB	Sentinel Lymph Node Biopsy
MRM	Modified Radical Mastectomy
SM	Simple Mastectomy
NST	Non-Specific Type
CI	Confidence Interval
IQR	Interquartile Range
PD-1	Programmed Cell Death Protein 1
TME	Tumor microenvironment
